# Autonomy-Supportive Faculty, Students' Self-System Processes, Positive Academic Emotions, and Agentic Engagement: Adding Emotions to Self-System Model of Motivational Development

**DOI:** 10.3389/fpsyg.2021.727794

**Published:** 2021-09-16

**Authors:** Maryam Bordbar

**Affiliations:** Department of Educational and Counseling Psychology, Ferdowsi University of Mashhad, Mashhad, Iran

**Keywords:** autonomy support, self-system processes, academic emotions, agentic engagement, faculty

## Abstract

The aim of this study was to investigate mediating roles of students' self-system processes and positive academic emotions in a relationship between supporting autonomy and agentic engagement. In This research structural equation modeling was used to analyze a conceptual model. The sample consisted of 452 undergraduate students of Ferdowsi University of Mashhad. The research instruments included the autonomy-supportive environment inventory, the self-system processes questionnaire, three questionnaires of academic emotions, and the agentic engagement scale. The findings showed that supporting autonomy had an indirect effect on students' achievement emotions, via self-system processes. Self-system processes had direct and indirect effects on agentic engagement, via positive academic emotions. Supporting autonomy had an indirect effect on agentic engagement by mediating role of self-system processes and positive academic emotions. Accordingly, emotions are proximal determinants of agentic engagement. Supporting autonomy and self-system processes affect agentic engagement from the pathway of academic emotions. Therefore, in addition to environmental factors and self-appraisals, it is necessary to consider students' emotional experiences to promote agentic engagement in learning settings.

## Introduction

The ultimate goal of any education system is to promote learners' academic assets (Skinner and Pitzer, 2012; Skinner et al., 2017). According to the ecological systems theory (Bronfenbrenner and Morris, 1998), the root of any development and achievement can be ascribed to complex, progressive and reciprocal interactions between an active, growing, bio-psychological organism (e.g., a learner) and people, objects and symbols (e.g., teachers, classmates, assignments, and goals) in its immediate environment (e.g., academic microsystem; school or university). These persistent forms of interactions are named proximal processes. These processes are a primary engine of individual achievement in the relevant microsystem, meaning that the individual evolves in that environment just through engagement in these interactions (Bronfenbrenner and Morris, 1998). Academic engagement, as far as the field of educational psychology is concerned, is considered as a proximal process, meaning that the only path to achieve assets (learning, good grades, resilience, etc.) in learning settings is engagement (Skinner and Pitzer, 2012; Skinner et al., 2017; Hiver et al., 2021).

Academic engagement is defined here as a powerful, directed and sustainable action. The three core features of this definition include being powerful, directed and sustainable, which are fundamental concepts in the area of motivation. This is because engagement is an outward manifestation of motivation (Skinner et al., 2009, 2017). Motivation is a fundamental source of energy, goal and sustainability, whereas engagement is the visible manifestation of them. Therefore, academic engagement refers to interactions of individual characteristics with important environmental characteristics and includes initiation of motivated action and its durability in the face of challenges and barriers. More specifically, academic engagement means learner's constructive, enthusiastic, willing, cognitively-focused participation in learning tasks and activities, which directly leads to positive academic performance (Skinner et al., 2009, 2017; Reeve et al., 2020a).

Scholars of academic engagement have taken into account different dimensions of academic engagement (Skinner et al., 2009, 2017), including the behavioral (effort and persistence), emotional (enthusiasm for learning and classroom) and cognitive (using effective cognitive strategies). The past few years have witnessed the emergence of a new dimension, which is agentic engagement (Reeve and Tseng, 2011). It refers to the fact that in addition to their behavioral and cognitive engagement, learners can also meaningfully contribute to teaching-learning processes. Not only do they try to learn, but they also seek to create motivationally a more supportive learning environment for themselves (Reeve et al., 2020b). Overall, agentic engagement is defined as learners' constructive contribution to the process of learning. Examples of agentic engagement embrace: expressing preferences, interests, and needs, asking questions, expressing attitudes, making suggestions and asking for elaboration (Reeve, 2013; Reeve et al., 2020b). It shares some common ground with other dimensions of engagement as it acts as a student-initiated pathway toward academic achievements. Notwithstanding this, significant differences can be observed and it is qualitatively different. As a matter of fact, agentic engagement is a unique proactive and a transactional form of engagement. Proactive suggests that learners, who demonstrate agentic engagement, may perform some actions before the process of learning begins (e.g., they ask their teacher). Transactional implies they negotiate with their teacher to construct a more motivationally-supportive learning environment (e.g., they speak with their teacher about how challenging, individual, satisfying or goal-congruent learning is). Of the diverse dimensions of engagement, the agentic dimension is the only one that counteracts the direct impact of environmental factors on achievement and explains the unique variance of success or achievement. This stresses that agency completely mediates the association between environment and positive educational assets (Reeve, 2013; Reeve et al., 2020b).

Given the importance of academic engagement and, in particular, the role of agentic engagement in yielding academic assets, some scholars of educational psychology have attempted to explain it. The sequence of *environment-self-action (engagement)* has been frequent in many models proposed in this regard (Connell and Wellborn, 1991; Skinner et al., 2009; Reeve, 2012; Reeve et al., 2020a). As pioneers in the field of education, Connell and Wellborn (1991), in their Self-System Model of Motivational Development maintain that the features of the environment (structure, participation, and autonomy support) determine academic engagement through self-system processes. Self-system processes are a set of appraisal processes through which individuals appraise their position in a given environment according to three basic psychological needs (competence, autonomy, and relatedness). These needs are organismic priorities around which the self-system is organized (Ryan and Deci, 2017). Competence refers to the need to experience oneself as capable of producing desired outcomes and avoid negative outcomes. Autonomy is the need to experience of choice in the initiation and maintenance of an activity. Relatedness is concerned with the need to feel connected with the social surrounding and the need to feel like a valuable person who is capable of love and respect. Self-system processes develop through interactions of individuals with their environments. When the psychological needs are met by such environments as schools and universities, student academic engagement can be boosted (Cheon and Reeve, 2015; Joe et al., 2017; Al-Hoorie, 2018; Patall et al., 2019; Reeve and Shin, 2020; Tirado-Morueta et al., 2020; Sökmen, 2021). Accordingly, inspired by the Self-Determination Theory (Deci and Ryan, 1985) and Self-System Model of Motivational Development (Connell and Wellborn, 1991), Skinner et al. (2009) put forward their theory of General Positive Motivational Development. In this coherent model, environmental variables (choice, structure, autonomy support, respect, etc.) shape different dimensions of engagement and bring about positive outcomes through self-perceptions (competence, task value, autonomy support, control beliefs, etc.).

In this way Reeve et al. (2004) offered the student-teacher dialectical framework. To better understand concepts of motivation and engagement within Self-Determination Theory, as the scholars put, it is essential to bear in mind that students have inner motivational resources (e.g., basic psychological needs) that allow them to be innately active and to be capable of engaging themselves constructively in leaning settings. Learning settings can offer meaningful opportunities wherein inner motivational resources are either supported (autonomy-supportive environment) or ignored (controlling environment). Therefore, learning environments affect students' motivation and engagement and the other way around (Reeve, 2012, Reeve et al., 2020a). In autonomy-supportive environment, autonomous students' motivations (interests, needs, preferences and personal goals) are supported (Assor et al., 2020). Three core characteristics of autonomy-supportive environment are provision of choice, provision of criticism and provision of goal/value/interest examination (Assor, 2012). Creating an environment wherein students can choose among different alternatives is provision of choice. Giving students a chance to express their agreement or opposing views in an empathetic, friendly and respectful environment is called provision of criticism. What is more, provision of goal/value/interest examination can be defined as presenting opportunities for students to engage in activities, tasks, experiences and discussions which allow them to assess and ponder their purposes, values and interests critically. The meaningful contribution of autonomy-supportive environment to academic engagement has been documented in several studies (Matos et al., 2018; Pineda-Báez et al., 2019; Benlahcene et al., 2020; Reeve et al., 2020a; Jiang and Zhang, 2021; Parker et al., 2021).

As observed in the models mentioned earlier, academic emotions that form an important part of learners' daily experiences (Wang et al., 2021) and effect agentic engagement have been ignored. On the other hand, the Control-Value Theory (Pekrun, 2006) explains academic emotions and introduces proximal antecedents of engagement as academic emotions. This may imply that academic emotions mediate the relationship between self-perceptions of the learning environment and academic engagement. In the latest version of Control-Value Theory, the Motivational-Emotional Model, Pekrun and Linnenbrink-Garcia (2014) presented various forms of academic emotions (achievement emotions such as hope, epistemic emotions such as curiosity, and social emotions such as empathy) and detailed their role in different dimensions of engagement. Research in this area has also highlighted the role of academic emotions in engagement (King et al., 2015; Zhen et al., 2017; Bordbar, 2019; Li et al., 2020; Yu et al., 2020; Zhang et al., 2020; Carmona-Halty et al., 2021).

**Figure 1:**
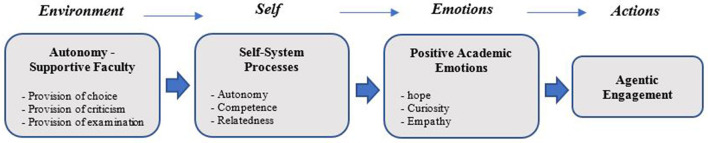
The conceptual model of current study extends Self-System Model of Motivational Development (Connell and Wellborn, 1991) together with consideration of Control-Value Theory (Pekrun and Linnenbrink-Garcia, 2012).

In an attempt to present a novel conceptual model, this study extends Self-System Model of Motivational Development (Connell and Wellborn, 1991), together with consideration of Control-Value Theory (Pekrun and Linnenbrink-Garcia, 2012). The present model (Figure 1) takes into account the sequence of *environment-self-emotion-action* and introduces positive academic emotions as proximal antecedents of agentic engagement. Hope is a positive and activating emotion that it is linked to student's future academic achievement (Pekrun and Linnenbrink-Garcia, 2014). Curiosity is the desire for the acquisition of new knowledge and information and generates positive feelings of intellectual interest and eliminates conditions of informational deprivation (Litman, 2019). Empathy is an emotional ability and refers to students' reactions to the observed experiences of another person in academic settings. It embraces emotional (ability to experience another person's emotions) and cognitive (ability to sense another person's emotions) reactions (Vossen et al., 2015).

Based on this conceptual model, it is expected that when the faculty provide their students with choice, criticism, and goal/value/interest examination opportunities, it will result in students' positive self-appraisals (perception of competence, autonomy, and relatedness). These appraisals can create positive emotional experiences in the learning environment. Ultimately, these emotional experiences will shape the agentic actions in students, actions that are the only path toward academic achievements and accomplishments.

## Materials and Methods

### Participants

The population for this investigation includes all undergraduates at Ferdowsi University of Mashhad in the first academic semester of 2019–2020. Following randomized multistage cluster sampling, 452 students (255 females and 197 males) were chosen as the research sample. Six faculties, including *Educational Sciences and Psychology* (*n* = 82), *Sciences* (*n* = 75), *Humanities and Literature* (*n* = 78), *Architecture* (*n* = 65), *Law* (*n* = 73) and *Agriculture* (*n* = 79), were chosen and from each faculty two classes were randomly picked out. All students of each class attended the survey, with an age range of 19 to 24 years.

### Questionnaires

#### The Agentic Engagement Scale

Reeve's (2013) Agentic Engagement Scale was used to measure students' agentic engagement. It has five-point Likert scale items, varying from 1 (totally disagree) to 5 (totally agree). To gauge the scale validity, convergent validity was used. The Academic Engagement Questionnaire score is significantly and positively correlated with the Psychological Needs Satisfaction Scale score (0.51) and the Academic Self-Efficacy Questionnaire score (0.48). Confirmatory factor analysis indicated that all items had a significant factor loading >0.47. Goodness-of-fit indices showed that the model fitted the data adequately, and Cronbach's alpha coefficient indicated high internal consistency (0.89).

#### Self-System Processes Questionnaire

To assess individual's basic needs satisfaction in general, Gagné's (Gagné, 2003) nine-item measure was used. This self-report scale measures to what extent individuals agree or disagree with items relevant to perceived autonomy, perceived competence and perceived relatedness. Items are rated on a five-point scale ranging from 1 (totally disagree) to 5 (totally agree). Confirmatory factor analysis showed that all items had a significant factor loading >0.40 and, like the original study, the items were loaded on their relevant factors. Goodness-of-fit indices indicated that the model fitted the data adequately, and Cronbach's alpha coefficient indicated acceptable internal consistency for the whole questionnaire (0.80).

#### The Autonomy-Supportive Environment Inventory

To assess faculty's autonomy-supportive behavior, three aspects, namely, provision of choice, provision of criticism and provision of goal/value/interest examination were investigated. The first two aspects were taken from autonomy-supportive environment inventory developed by Assor et al. (2002) and the last one from Assor's (Assor, 2012) Goal/Value/Interests Examination Support Scale. Each scale has 7 five-point Likert scale items, ranging from 1 (totally disagree) to 5 (totally agree). For this study, confirmatory factor analysis suggested that all items had a significant factor loading >0.40, and, like the original study, the items were loaded on their relevant factors. Goodness-of-fit indices indicated that the model fitted the data adequately, and Cronbach's alpha coefficients were reported as 0.89 for provision of choice, 0.81 for provision of criticism and 0.85 for provision of goal/value/interest examination.

#### The Subscale Hope From Academic Emotions Questionnaire

The subscale hope was taken from Pekrun et al.'s (Pekrun et al., 2002) academic emotions questionnaire. This scale has three subscales of class-related hope, learning-related hope and test-related hope. For the present study, the two sub-scales of learning-related hope (with six items) and class-related hope (with eight items) were utilized and the items are assessed on a five-point Likert scale, ranging from 1 (totally disagree) to 5 (totally agree). Confirmatory factor analysis for this paper indicated that all items had a significant factor loading >0.48 and, like the original study, the items were loaded on their relevant factors. Goodness-of-fit indices indicated that the model fitted the data adequately, and Cronbach's alpha coefficients were reported as 0.90 for class-related hope and 0.89 for learning-related hope.

#### Epistemic Curiosity Questionnaire

This scale was developed by Litman et al. (2010) to assess epistemic curiosity and has 10 items. It is scored on a four-point Likert scale, ranging from 1 (almost never) to 4 (almost always). The reliability of the original scale is 0.75 and its validity was measured using convergent validity and discriminant validity (Litman et al., 2010). Epistemic curiosity questionnaire score is significantly and positively correlated with the intrinsic motivation inventory score (0.36) and is significantly and negatively correlated with the extrinsic motivation inventory score (−0.15). In this research, Goodness-of-fit indices indicated that the model fitted the data adequately, and the whole questionnaire enjoyed a high internal consistency (0.87).

#### The Empathy Subscale From Empathy and Sympathy Inventory

This 12-item questionnaire was developed by Vossen et al. (2015) and measures empathy and sympathy. It is scored on a five-point Likert scale, ranging from 1 (never) to 5 (always). The subscale empathy has eight items and its original reliability is 0.86, and test–retest reliability for the original measure was 0.66 with a 2-week interval. Vossen et al. (2015) used internal consistency, convergent validity and discriminant validity to measure validity. The empathy questionnaire score is significantly and positively correlated with the perspective-taking questionnaire score (0.49) and is significantly and negatively correlated with the aggression questionnaire score (−0.45). In current research Confirmatory factor analysis showed that all items had a significant factor loading >0.41 and, Goodness-of-fit indices indicated that the model fitted the data adequately, and Cronbach's alpha coefficients indicated acceptable internal consistency for the questionnaire (0.85).

### Procedure and Data Analysis

Prior to the survey, the participants completed an informed consent form to participate in the study and they were assured that their information would be confidential and participation is entirely voluntary. Participants were then asked to complete the questionnaires to collect the necessary data. Each questionnaire took ~25 min to complete.

The present research follows Structural Equation Modeling (SEM) to analyze the relationships between variables in the proposed model. The data analysis was guided by Descriptive Statistics (Mean and Standard Deviation) in SPPSS (version 21). Also, AMOS (version 21) was utilized to perform SEM for the analysis of structural relationships between variables of the model and path coefficients. To find the mediation effects of mediator variables, the bootstrapping method was used.

## Results

To check the assumptions of SEM, data screening was utilized for normality, outliers, and missing values. Univariate normality was assessed by skewness, kurtosis and Q-Q plot, and multivariate normality was measured by Mardia's coefficients in AMOS. Skewness and Kurtosis values for all observed variables are < +1 indicating that the distribution of all observed variables is not significantly different from the normal distribution. Additionally, Mardia's coefficient showed a multivariate normal distribution. Examination of the normal probability plot of the observed variables revealed that the points are closer to the diagonal line enjoying a normal distribution of the variables. In order to check the linearity of the relationship between the research variables, the residual scatterplot and scatterplot matrices were examined. In the matrix of scatterplots, all plots were approximately elliptical and showed the linearity of the relationship between the observed variables. The residual scatter plot was not curved and was distributed in a rectangular shape around the zero residual value, indicating a linear relationship between the predicted dependent variable scores and the predicted errors. Multi-collinearity of predictor variables was also examined using tolerance statistics and variance inflation factor. The results showed that the tolerance values obtained for the variables are above 0.01, which indicates the absence of multi-collinearity of the variables. Also, the amount of variance inflation factor obtained for the variables was <10, which indicates that there is no multi-collinearity of the variables.

The descriptive statistics can be seen in Table 1 which gives a snapshot of the research variables. To analyze the mediating role of self-system processes and academic emotions in the relationship between faculty's support of autonomy and agentic engagement, initially goodness of fit indices and then regression weights and coefficients of the structural relationship of latent variables in the model were analyzed. In order to find a better model fit, the insignificant path in the model was removed and then modification indices were taken into account. The AMOS proposed some modifications, including the calculation of covariance among errors of observed variables. Goodness-of-fit indices are reported in Table 2. After a round of modifications, goodness-of-fit indices suggested that the model fitted the data perfectly.

**Table 1:** Mean and standard deviation of research variables.

**Variables**	**Mean**	**SD**
Provision of choice	21.52	5.12
Provision of criticism	22.91	4.51
Provision of examination	23.24	4.12
Autonomy	11.12	2.52
Competence	12.32	1.92
Relatedness	12.14	2.01
Hope	54.51	9.20
Curiosity	29.50	7.13
Empathy	26.72	4.11
Agentic engagement	19.53	3.92

**Table 2:** Model fit indices.

**Fitness Index**	**X^**2**^/df**	**GFI**	**AGFI**	**CFI**	**NFI**	**IFI**	**TLI**	**RMSEA**	**PCLOSE**
Amount	2.50	0.96	0.96	0.96	0.97	0.97	0.95	0.05	0.30

**Figure 2:**
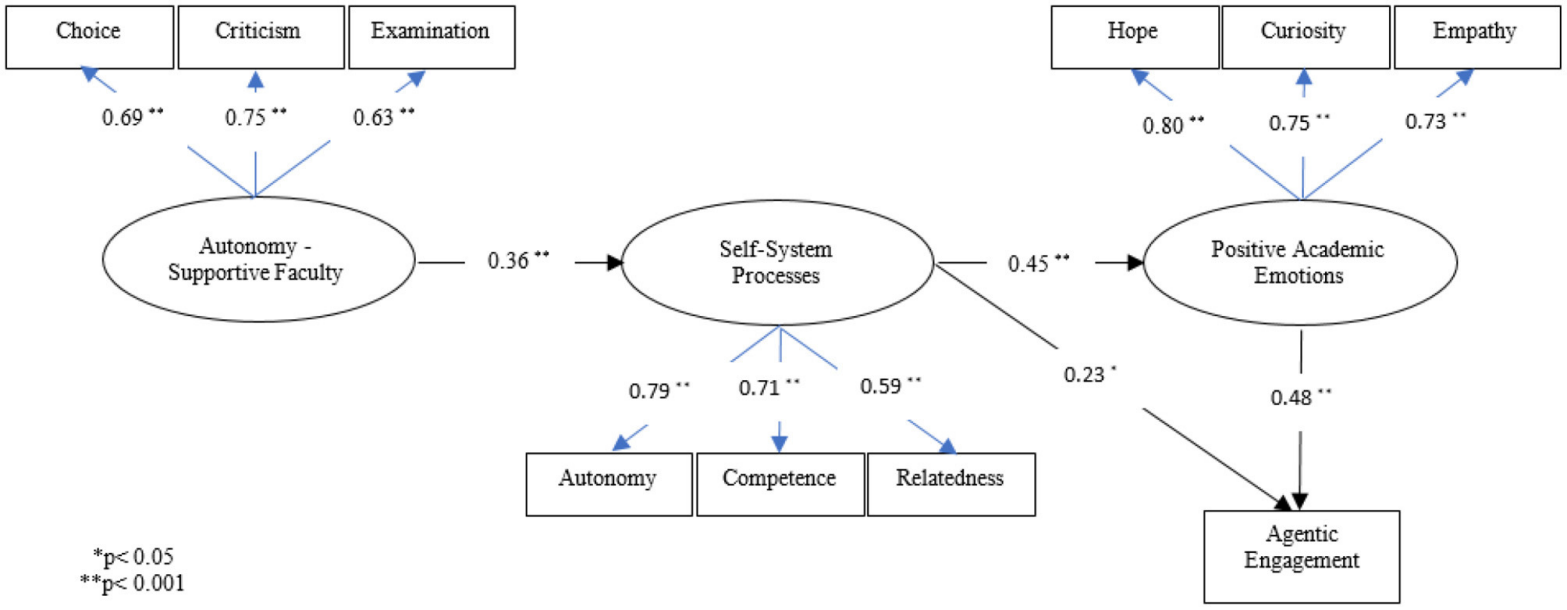
Standardized regression weights in model.

The regression weights of three measurement models were significant (Figure 2), suggesting that all indices were determinants of the relevant latent variables. Of the three indices of faculty's support of autonomy, provision of criticism had the highest weight in defining the latent variable; in other words, the most powerful index. As to the latent variable of self-system processes, perceived autonomy was the most powerful index. Finally, hope was the most powerful index of academic emotions.

The outputs of structural model analysis, namely, the direct effects of latent variables in the model and the indirect effects—using bootstrapping and with a 95% confidence interval—are shown in Table 3. As Table 3 shows, the variable of faculty's support of autonomy has a significant and direct effect on self-system processes. This variable has a significant and indirect effect on agentic engagement, via self-system processes and academic emotions. Also, this variable has a significant and indirect effect on academic emotions, via self-system processes. Self-system processes have a significant and direct effect on agentic engagement. In addition, the self-system processes have an indirect effect on agentic engagement, via academic emotions. Overall, it can be concluded that these self-system processes and academic emotions play a mediating role between faculty's support of autonomy and agentic engagement, and the research model accounts for 51% of students' agentic engagement variance.

**Table 3:** Direct, indirect and total effects in model.

**Path**		**Direct effect**	**Indirect effect**	**Total effect**	**Explained Variance**
To agentic engagement	From autonomy Support	—	0.31^**^	0.31^**^	51%
	From self-system processes	0.23^*^	0.32^**^	0.55^**^	
	From academic emotions	0.48^**^	—	0.48^**^	
To academic emotions	From autonomy support	—	0.26^**^	0.26^**^	39%
	From self-system processes	0.45^**^	—	0.45^**^	
To self-system processes	From autonomy support	0.36^**^	—	0.36^**^	13%

* *P < 0.05*

** *P < 0.001*.

## Discussion

This research aimed to investigate the mediating role of students' self-system processes and academic emotions in the relationship between autonomy-supportive faculty and students' agentic engagement. The results suggested that two paths in the final model determined agentic engagement: *environment-self-agentic engagement* and *environment-self-emotion-agentic engagement*. Faculty's autonomy support explain students' agentic engagement variance via self-system processes. Also, faculty's autonomy support and self-system processes explain agentic engagement via academic emotions.

As to the mediating roles of self-system processes in the relationship between autonomy-supportive faculty and agentic engagement, the initial models showed the path *environment-self-agentic engagement* (Connell and Wellborn, 1991; Skinner et al., 2009; Reeve, 2012). Numerus studies have confirmed this path (Reeve and Shin, 2020; Tirado-Morueta et al., 2020). According to organismic approaches, learners and more generally humans are active agents and can effectively engage themselves in the (learning) environment (Reeve, 2013; Ryan and Deci, 2017). Engagement in the environment has root in intrinsic motivation (such as basic psychological needs). Autonomy supportive environments offer opportunities for learners which can result in vitalizing their intrinsic motivation. This motivation stands out in the action and learner's agentic engagement in learning activities and tasks become apparent (Molinari and Mameli, 2018; Cohen et al., 2020).

Therefore, faculty, who support autonomy, can create a positive sense of self in students through addressing their basic needs. Such faculty promote a sense of competence, autonomy and relatedness in students by encouraging them to choose learning activities, listening to their ideas, accepting their criticism, and giving them a chance to reflect on important life issues, attitudes, values, and concerns. Within this learning environment, students perceive themselves as capable of attaining desired outcomes and reaching goals (perception of competence). They have a sense of independence in choosing learning activities and see a harmony between sources of intrinsic motivation and their activities (perception of autonomy). They experience a sense of being loved, valued and respected by others (perception of relatedness). Taken together, positive appraisal of self leads to agentic engagement in activities (Matos et al., 2018; Jiang and Zhang, 2021).

Also the second path, *environment-self-emotion-agentic engagement*, was confirmed. This means that academic emotions are proximal antecedents of agentic engagement. The previous theories suggests that experiencing positive emotions can lead to disengagement. This is because when the individual experiences positive emotions, they feel that they are moving fast enough to meet their goals, and consequently, further engagement deems unnecessary for them (Carver et al., 1996). Or, the experience of positive emotions is a sign for them that everything is fine and there is no need to further engage in activities (Schwartz and Clore, 1996). However, Pekrun and Linnenbrink-Garcia (2014) are of the view that these theories have ignored various aspects of value and activation of academic emotions and also object of academic emotions. Activating emotions like hope, increase engagement and when the object of emotion is learning task, academic emotion is a facilitator and a proximal antecedent of agentic engagement. Some have even called it a catalyst for engagement (King et al., 2015). The role of emotions in engagement have confirmed in studies (King et al., 2015; Zhen et al., 2017; Bordbar, 2019; Li et al., 2020; Yu et al., 2020; Zhang et al., 2020; Carmona-Halty et al., 2021).

On the other hand, the experience of positive emotions in educational environments can generally expand thought-action repertoires (Fredrickson, 2013). Experiencing positive emotions increases approach-oriented behaviors that lead to engagement in activities, while the experience of negative emotions during the task limits thought-action repertoires. When learners' mindsets are limited to avoidant behavior, agentic engagement is seriously challenged. Expansion of thought-action repertoires while experiencing positive emotions has another important function that is building personal resources. These resources can be used in subsequent activities. Indeed, agentic engagement is considered as a kind of personal resource that can facilitate further learning in the environment. Therefore, the experience of positive emotions not only facilitates momentary agentic engagement, but also plays an effective and positive role in the learner's subsequent engagements in learning tasks and activities.

Experiencing positive academic emotions such as curiosity can effectively maintain students' focus on assignments and leads to knowledge-seeking behaviors and promotes agentic engagement in learning settings (Vracheva et al., 2020). Experiencing activating positive emotions like hope will create a positive sense of future academic accomplishments (Tomás et al., 2020). With such a sense of hope, the students constructively engaged in the learning environment and can make their way toward achievements and goals through agentic actions. Also, learning in educational settings is an interactive process and engagement means the interaction of individual characteristics with environmental characteristics. Indeed, empathetic relationships and the experience of social emotions like empathy throughout the learning activity create a motivational force driving students toward more agentic engagement in the environment (Pekrun and Linnenbrink-Garcia, 2012).

On the other hand, the role of the environment and self-appraisals in emotional experiences has been highlighted in theories (Lazarus, 1994). Broadly speaking, environmental features determine emotional experiences through self-appraisals. These appraisals of environment based on personal motivations (intrinsic or acquired) lead to emotion. When personal motivations are given due attention in the environment, emotions emerge.

Also according to the self-determination theory (Ryan and Deci, 2017), supportive learning environment can yield positive emotion outcomes for learners. This association is such that the characteristics of supportive learning environment through interactions with the inherent psychological needs of the students create a psychological power in them which is their positive appraisal and perception of competence, autonomy and relatedness. This power can create positive emotions. Ultimately, the positive emotions push students to express their interests and needs, ask questions, seek clarification and elaboration and offer suggestions to their teachers for better learning. More specifically, they should play a constructive role in changing their learning environment.

Theoretically, the present study was a significant contribution to *Self-System Model of Motivational Development* (Connell and Wellborn, 1991) as emotional experiences were introduced. This study highlighted the role of academic emotions, which are an integral part of learning environments. In light of the findings, the study has some educational implications. Faculty should focus on a university environment which gives support to autonomy and they can set the stage for the students to choose their course content, to criticize, to evaluate their goals, values and interests. This helps students engage more constructively in activities. Teachers should pay attention to students' self-system processes, which is their perception of competence, autonomy and relatedness as significant factors in academic engagement. They should assess students' perception of self and should design interventions to promote these perceptions. They should also focus on students' academic emotions throughout the learning activities as they are proximal antecedents and predicting of agentic engagement. Ignoring students' experience of emotions can lead waste to teachers' efforts to strengthen their students' motivations (self-appraisals). This is because positive academic emotions can provide the ground for students' engagement, and consequently, their accomplishments at university.

Given that the present research was quantitative, it is suggested that future studies examine the proposed model in a mixed-methods research design. The use of the embedded research design wherein the researcher embeds a qualitative component or data within quantitative research to examine the underlying mechanisms relevant to research variables is recommended. This should be noted that the research was a cross-sectional study and future studies are recommended to examine research variables in a longitudinal design throughout a semester (at beginning, middle and end of the semester). In the longitudinal design, transpositions and interactions between variables can be analyzed in detail.

## Data Availability Statement

The raw data supporting the conclusions of this article will be made available by the authors, without undue reservation.

## Ethics Statement

The study involves human participants. They were assured that their information would be confidential and the participation was entirely voluntary. They provided their written informed consent to participate in the study.

## Author Contributions

The author confirms being the sole contributor of this work and has approved it for publication.

## Conflict of Interest

The author declares that the research was conducted in the absence of any commercial or financial relationships that could be construed as a potential conflict of interest.

## Publisher's Note

All claims expressed in this article are solely those of the authors and do not necessarily represent those of their affiliated organizations, or those of the publisher, the editors and the reviewers. Any product that may be evaluated in this article, or claim that may be made by its manufacturer, is not guaranteed or endorsed by the publisher.
